# Irradiation Attenuates Systemic Lupus Erythematosus-Like Morbidity in NZBWF1 Mice: Focusing on CD180-Negative Cells

**DOI:** 10.1155/2023/9969079

**Published:** 2023-10-18

**Authors:** Kazuko Fujita, Taku Kuwabara, Bing Wang, Kaoru Tanaka, Kei Ito, Yuri Akishima-Fukasawa, Tetuo Mikami, Yoshikiyo Akasaka, Toshiharu Ishii

**Affiliations:** ^1^Department of Pathology, School of Medicine, Toho University, Ota-Ku, Tokyo 143-8540, Japan; ^2^Department of Molecular Immunology, School of Medicine, Toho University, Ota-Ku, Tokyo 143-8540, Japan; ^3^Institute for Radiological Science, National Institutes for Quantum Science and Technology, Chiba-City, Chiba 263-8555, Japan; ^4^Department of Medical Technology, Faculty of Health Sciences, Tsukuba International University, Ibaragi, Tsuchiura-City 305-8577, Japan; ^5^Department of Pathology, Saiseikai Yokohamashi Tobu Hospital, Kanagawa, Yokohama-City 230-8765, Japan

## Abstract

Systemic lupus erythematosus (SLE) is a chronic autoimmune disease characterized by the production of autoantibodies that can induce systemic inflammation. Ultraviolet-A and X-ray irradiation have been reported to have therapeutic effects in patients with SLE. We previously demonstrated that CD180-negative cells, these are radiosensitive, contribute to the development of SLE-like morbidity in NZBWF1 mice. In this study, the effects of irradiation on SLE-like morbidity manifestations in NZBWF1 mice and on CD180-negative cells were investigated. Whole-body irradiation, excluding the head, attenuated SLE-like morbidity *in vivo*, as indicated by the prevention of the renal lesion development, inhibition of anti-dsDNA antibody production, reduction of urinary protein levels, and prolongation of the lifespan. Irradiation also reduced the proportion of CD180-negative cells in the spleen. Although other immune cells or molecules may be triggered because of the whole-body irradiation treatment, previous research, and the current results suggest a strong relationship between the radiation-induced decrease in CD180-negative cells and the amelioration of SLE-like morbidities. Clinical trials assessing CD180-negative cells as a therapeutic target for SLE have been hampered by the lack of validated cell markers; nonetheless, the present findings suggest that radiotherapy may be a new therapeutic strategy for managing SLE symptoms.

## 1. Introduction

Systemic lupus erythematosus (SLE) is a chronic autoimmune disease characterized by the production of autoantibodies that bind to self-antigens and deposited within tissues of the host, which consequently results in widespread systemic inflammation. Ultraviolet-A irradiation therapy has been reported to improve the manifestations of SLE activity without significant toxicity [1–5]. Moreover, anticancer radiation therapy has also been reported to alleviate SLE symptoms [6–10]. Possible explanations for the mechanism undergoing the symptomatic improvements after these irradiation treatments include modulates T helper (Th) 1/Th2 and cytotoxic T (Tc) 1/Tc2 balance in patients with SLE [11], apoptosis of B cells [12], and reduction of natural killer cells [13]. However, radiotherapy for malignant thymoma or lymphoma has also been reported to trigger SLE development, which suggests a relationship between the thymus and SLE etiology [14, 15]. A previous study showed that treatment with the nonenzymatic antioxidants *N*-acetylcysteine and cysteamine significantly reduced the mortality rate of NZBWF1 mice, which is a preclinical model of human SLE [16]. Another study reported that total lymphocyte (TL) irradiation can prolong the survival of these mice [17]. Moreover, TL irradiation therapy resulted in the resolution of the autoimmune symptoms in MRL/Mp-+/+ (MRL/n) and MRL/Mp-lpr/lpr (MRL/l) SLE murine models [18]. Currently, human SLE treatments include conventional immunosuppressive drugs, such as corticosteroids and immunomodulators, which are novel biological agents that target molecules of the adaptive and innate immune systems [19–21].

CD180, which is also known as radioprotective 105-kDa protein (RP105), is a homolog of the Toll-like receptor (TLR)-4 and is a key regulator of *in vitro* B-cell proliferation and death in response to various stimuli [22–28]. Previous studies have demonstrated that, in contrast to CD180-negative cells, CD180-positive B cells are radioprotective [22, 29]. Clinical investigations have shown that CD180 is involved in TLR-7/9-mediated B-cell activation in patients with chronic lymphocytic leukemia [30], common immunodeficiency [31], and relapsing-remitting multiple sclerosis [32]. Moreover, CD180 has been reported to be associated with autoimmune central nervous system disorders and to regulate the activity of dendric cells and monocytes in patients with SLE [33]. Patients with autoimmune diseases, such as SLE, Sjögren's syndrome, and dermatomyositis, were reported to have a higher proportion of CD180-negative cells in the peripheral blood than healthy individuals [34–36]. In addition, the percentage of CD180-negative cells can change based on the level of disease activity [37, 38]. Aging has also been previously reported to be correlated with an increased proportion of CD180-negative B cells in the spleen of NZBWF1 mice. During this process, CD180-negative B cells were found to infiltrate the renal lesions and promote their progression, as well as to produce and secrete autoantibodies into circulation, such as anti-dsDNA and anti-histone antibodies [39]. Hence, these findings indicate that CD180-negative cells contribute to the development of SLE-like morbidity in NZBWF1 mice.

In this study, the effects of whole-body irradiation on SLE-like morbidity in NZBWF1 mice were investigated based on renal lesion development, urinary protein levels, anti-dsDNA antibody production, and lifespan were evaluated to determine whether whole-body irradiation could ameliorate SLE-like morbidity and how CD180-negative cells could contribute for this process *in vivo*. The results of this study are expected to provide new foundations for the development of therapeutic strategies to eliminate autoantibodies and prevent SLE development.

## 2. Materials and Methods

### 2.1. Animals

Eleven-week-old female NZBWF1 mice were purchased from Shizuoka Laboratory Corporation (SLC, Hamamatsu, Japan) and maintained in a conventional animal facility in a 12/12-hr light/dark cycle (lights on from 08:00 to 20:00). After 1 week of acclimatization, the mice were tested for urinary proteins and their body weights were measured daily until SLE-like morbidity onset; afterward, urinary protein and body weight were assessed weekly. Group A, consisted of 13 paired mice, was euthanised at 15 weeks after irradiation or sham-irradiation. The mice were sacrificed by CO_2_ asphyxiation, followed by cervical dislocation, according to guidelines of the Toho University Care and User Committee and the Institutional Animal Care and Use Committee of the National Institute of Radiological Sciences, National Institutes for Quantum Science and Technology (QST-NIRS). Group B, consisted of 50 paired mice, was maintained alive until natural death. All experimental protocols involving the mice were reviewed and approved by the Toho University Care and User Committee (No. 81, 17, 10-54-55, 11-55-55, 12-31-184) and by QST-NIRS (No. 07-1001-3).

### 2.2. Urinary Protein Levels

Proteinuria levels were assessed using a test paper (Pretest 3aII, FUJIFILM Wako Pure Chemical Industries, Osaka, Japan). This colorimetric assay is specific for albumin and estimates urinary protein at levels within five levels as follows: (0) 0 mg/dL of protein, (1) 30 mg/dL of protein, (2) 100 mg/dL of protein, (3) 300 mg/dL protein, and (4) 1,000 mg/dL of protein.

### 2.3. Criteria for Disease Onset and Mice Pairing

Disease onset was established based on urinary protein trends without performing an invasive procedure, in agreement with the previously reported correlation between increased urinary protein and kidney injury [39]. The mouse was judged as disease onset when it displayed urinary protein levels of 1+ or higher for 3 days within 5 continuous days.

After the judgment of disease onset, the animals were paired timely based on body weight. The pairing in this study was performed to increase the accuracy of the comparisons since NZBWF1 mice show greater individual variations in the onset of SLE symptoms.

### 2.4. Irradiation

X-rays were generated by a Pantak-320S X-ray Apparatus (Shimadzu Industrial Systems Co., Ltd., Otsu, Japan) operated at 200 kVp and 20 mA and using 0.50 mm Al + 0.50 mm Cu filter. After the judgment of disease onset, one of each paired mice was randomly selected and was subjected to whole-body irradiation (except the head) at a dose of 4 Gy. The control mice of each pair were sham-irradiated. An exposure rate meter (AE-1321 M, Applied Engineering Inc., Tokyo, Japan) with an ionization chamber (C-110, 0.6 mL, JARP, Applied Engineering Inc., Tokyo, Japan) was used for the dosimetry. The dose rate for delivering the irradiation protocol was approximately 0.50 Gy/min.

### 2.5. Histopathological Grading of Renal Amounts

To evaluate renal lesions, the kidneys from mice 15 weeks after irradiation or sham irradiation were removed, fixed in 10% formalin for 7 days and embedded in paraffin. The paraffin blocks were cut into 3-mm sections, which were then mounted and stained with hematoxylin and eosin (HE) staining and periodic acid Schiff (PAS) staining for histopathological observation, according to standard protocols [40, 41]. Based on the histological staining results, the extent of SLE-like glomerular lesion development in each NZBWF1 mouse was classified into six grades, as follows: Grade 0, normal glomeruli; Grade 1, focal distribution (less than 50%) of glomeruli with mild mesangial hypercellularity; Grade 2, focal distribution of glomeruli with moderate mesangial hypercellularity; Grade 3, diffuse distribution of glomeruli (more than 50%) with moderate mesangial hypercellularity; Grade 4, diffuse distribution of glomeruli with focal active lesions, such as wire loop lesions, or exudative changes; and Grade 5, diffuse glomerular sclerosis with decreased number of glomerular capillaries.

### 2.6. Quantitative Analysis of Autoimmune Antibody Levels

To measure autoantibodies, peripheral blood samples were collected from NZBWF1 mice 6, 12, 15, and 36 weeks after irradiation or sham irradiation, and they were evaluated for anti-dsDNA antibody levels using enzyme-linked immunosorbent assay kits (Alpha Diagnostic, San Antonio, TX, USA), according to the manufacturer's instructions.

### 2.7. Flow Cytometric Analysis

Single-cell suspensions were prepared according to the following protocol. Briefly, splenocytes from the NZBYF1 mice were collected 15 weeks after irradiation or sham irradiation and were suspended in a lysis buffer, phosphate buffer solution containing 150 mM NH_4_Cl, to eliminate erythrocytes. The cell suspension was treated with an Fc blocker, anti-CD16/CD32 (BD Bioscience, San Jose, CA, USA). The cells were then stained with APC-conjugated anti-CD3 (BD Biosciences), FITC-conjugated anti-B220 (BD Biosciences), and PE-conjugated anti-CD180 (BioLegend, San Diego, CA, USA) antibodies, and were subsequently analyzed using a FACS Aria Ⅲ system (BD Bioscience). An isotype control was used for each antibody.

### 2.8. Statistical Analysis

Statistical evaluation of the data of the paired irradiated and sham-irradiated mice was performed using paired t-tests to assess the population of CD-180 negative cells, amount of anti-dsDNA antibody 15 weeks after treatment, and length of survival period. Student's *t*-test was performed to assess differences in the amounts of anti-dsDNA antibody 6, 12, and 36 weeks after treatment. The Wilcoxon signed-rank sum test was performed to assess the glomerular lesion grade and urinary protein levels. The Kaplan–Meier method was used to determine the survival rate of the NZBWF1 mice. Differences were considered statistically significant at *p* < 0.05.

## 3. Results

### 3.1. Mouse Data

At the onset of SLE-like morbidity, the average body weight in Group A mice was 27.6 g (standard deviation: SD: 1.31), and that in Group B mice was 28.2 g (SD: 1.37). The mean period from the start of the experiment to the onset of the disease in Group A and Group B were 11.3 days (SD: 0.62) and 13.2 days (SD: 2.40), respectively. The mean difference in disease onset date between paired mice in Group A was 0.63 days (SD: 0.66), whereas that in Group B was 0.42 days (SD: 0.64). No significant difference between Groups A and B (*p*=0.058) was recognized. The mean period from disease onset to sham irradiation or irradiation in Group A was 1.69 days (SD: 0.62, range: 0–2 days) and that in Group B was 0.64 days (SD: 0.85, range: 0–2 days).

### 3.2. Irradiation Prevents the Histopathological Development of Renal Lesions

**Figure 1:**
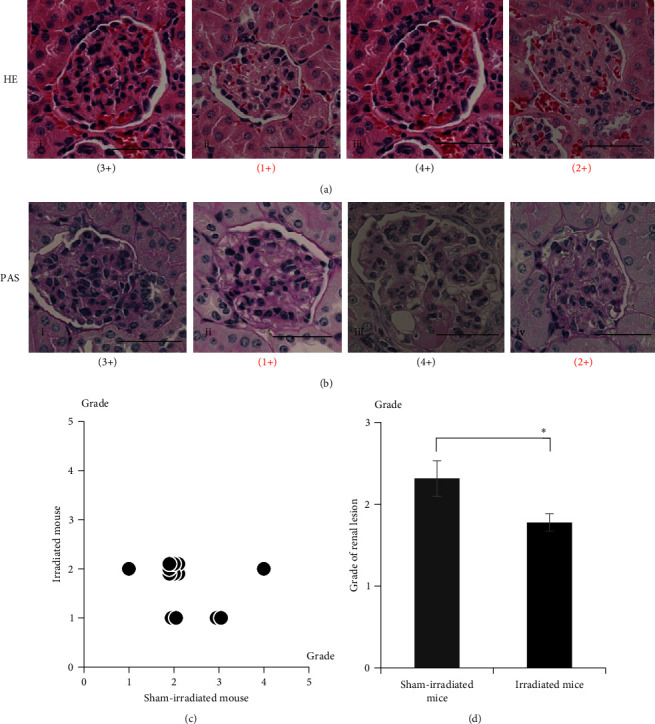
Irradiation prevents the histopathological development of renal lesions in NZBWF1 mice. (a, b) Histopathological assessment of kidney tissue samples from paired mice 15 weeks after treatment based on hematoxylin and eosin (HE) staining (a) and periodic acid-Schiff (PAS) staining (b) (Group A). Images of i and iii, and ii and iv were from sham-irradiated and 4 Gy-irradiated mice, respectively. Images of i and ii, and iii and iv were from paired mice, respectively. Scale bars indicate 50 *μ*m. (c) Comparison of the renal lesion grades between paired sham-irradiated and irradiated mice 15 weeks after treatment (*n* = 13 paired mice). (d) Renal lesion grades in sham-irradiated and irradiated mice 15 weeks after treatment (*n* = 13 in each group). Open and solid bars indicate sham-irradiated and four irradiated mice, respectively. Data are presented as mean ± standard error. The  ^*∗*^ presents significant differences at 0.01 < *p* < 0.05.

To examine the effects of irradiation on SLE-associated renal lesions, histopathological analyses were performed on the kidney tissue samples of paired mice 15 weeks after treatment. Lesion grades 1–4 were detected by using HE staining (Figure 1(a)) and PAS staining (Figure 1(b)). Grade 5 lesion was not observed in any experimental mice.  Figure 1(c) shows a comparison of the renal lesion grades in paired mice. Overall, the lesion grades were markedly lower in the irradiated mice than in their paired sham-irradiated mice (*p*=0.378, Figure 1(c)), which was noticeable by the significant difference in the mean lesion grade of the two groups (sham-irradiated mice: 2.31, irradiated mice: 1.77, *p*=0.035) (Figure 1(d)). Hence, irradiation treatment can prevent the histochemical developments of SLE-related renal lesions *in vivo*.

### 3.3. Irradiation Decreases Urinary Protein Levels

**Figure 2:**
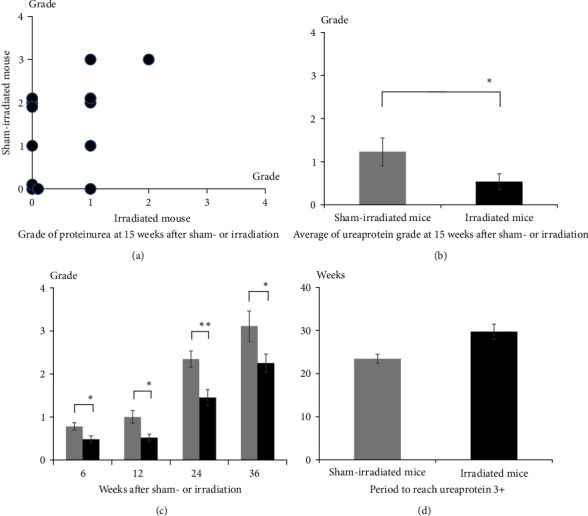
Irradiation prevents proteinuria levels in NZBWF1 mice. (a) Comparison of the urinary protein levels between paired sham-irradiated and 4 Gy-irradiated mice 15 weeks after treatment (*n* = 13 paired mice, Group A). (b) Urinary protein levels in sham-irradiated and irradiated mice. Open and solid bars indicate sham-irradiated and 4 Gy-irradiated mice, respectively (*n* = 13 in each group, Group A). (c) Urinary protein levels at 6 (*n* = 50 in each group), 12 (*n* = 49 vs. 50), 24 (n = 35 vs. 49), and 36 (n = 9 vs. 24) weeks after treatment (Group B) are presented as the mean value ± standard error. (d) Duration from sham-irradiation or irradiation to the development of level 3+ urinary protein (*n* = 50 in each group, Group B). Open and solid bars indicate sham-irradiated and irradiated mice, respectively. Each group consisted of 50 mice (Group B). Data are presented as mean± standard error. The  ^*∗*^ presents significant difference at 0.01 < *p* < 0.05 and  ^*∗∗*^0.001 < *p* < 0.01.

To elucidate the therapeutic effects of irradiation, we examined the urinary protein levels. Urinary protein levels were significantly lower in irradiated mice than in sham-irradiated mice 15 weeks after treatment by comparison with paired mice (Figure 2(a)) and with mean levels (sham-irradiated mice: 1.23, irradiated mice: 0.538, *p*=0.022; Figure 2(b)). Since it has been reported that urinary protein levels increase with time after SLE onset [39], a time course study was performed 6, 12, 24, and 36 weeks after treatment (Figure 2(c)). Urinary protein levels increased with time after SLE onset in both irradiated and sham-irradiated mice; however, protein levels were markedly lower in irradiated mice than in sham-irradiated mice at all time points. Mean protein levels of sham-irradiated mice and irradiated mice were 0.78 and 0.48 at 6 weeks (*p*=0.0138), 1.0 and 0.52 at 12 weeks (*p*=0.00547), 2.34 and 1.45 at 24 weeks (*p*=0.00163), 3.11 and 2.25 at 36 weeks after treatment (*p*=0.00419), respectively. Detailed analysis of the duration from onset until the development of level 3+ protein further showed that it was markedly longer in irradiated mice (23.4 weeks after treatment) than in sham-irradiated mice (29.7 weeks after treatment) (*p*=0.000960; Figure 2(d)). These results indicate that irradiation decreases SLE-related urinary protein levels.

### 3.4. Irradiation Inhibits Anti-dsDNA Antibody Production

**Figure 3:**
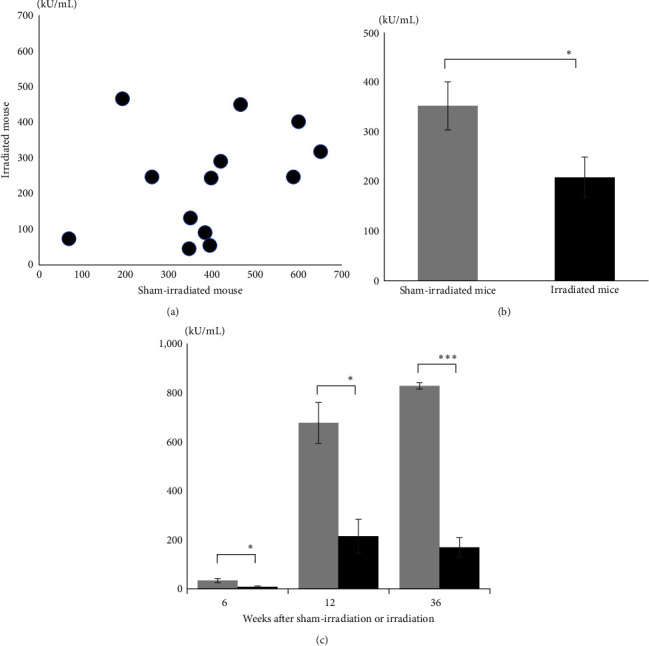
Irradiation suppresses the production of anti-dsDNA autoantibody in NZBWF1 mice. (a) Comparison of anti-dsDNA antibody titers in peripheral blood between paired sham-irradiated and 4 Gy-irradiated mice 15 weeks after treatment (*n* = 13 pairs, Group A). (b) Anti-dsDNA antibody titers in the peripheral blood between paired sham-irradiated and 4 Gy-irradiated mice 15 weeks after treatment (*n* = 13 pairs, Group A). Open and solid bars indicate sham-irradiated and irradiated mice, respectively. Data are presented as the mean ± standard error. (c) Anti-dsDNA antibody mean titers in the peripheral blood from sham-irradiated and four irradiated mice at 6 (*n* = 8 in each group), 12 (*n* = 6 in each group), and 36 (*n* = 3 vs. 4, respectively) weeks after treatment. Open and solid bars indicate, respectively (Group B). Data are presented as the mean ± standard error.  ^*∗*^0.01 < *p* < 0.05 and  ^*∗∗∗*^*P* < 0.001.

Since autoantibodies, such as anti-dsDNA and anti-histone antibodies [33], make a role of SLE-like morbidity in NZBWF1 mice, we evaluated the anti-dsDNA antibody titers in the peripheral blood of paired mice 15 weeks after treatment (Figure 3(a)). Notably, the anti-dsDNA antibody production was more strongly inhibited in the irradiated mice than in the sham-irradiated mice. Average titers of the anti-dsDNA antibody in sham-irradiated mice and irradiated mice were 352.5 and 208.3 kU/mL, respectively; *p*=0.0159; Figure 3(b)). A time course study of anti-dsDNA antibodies was performed 6, 12, and 36 weeks after treatment (Figure 3(c)). The mean anti-dsDNA antibody titers of sham-irradiated mice and irradiated mice were 33.4 and 8.01 kU/mL at 6 weeks (*p*=0.017), 676.3 and 214.2 kU/mL at 12 weeks (*p*=0.0052), 827.4 and 168.4 kU/mL at 36 weeks after treatment (*p*=0.00092), respectively. Anti-dsDNA antibody levels increased in both groups compared with pretreatment levels, whereas they were lower in irradiated mice at all time points compared with those in the sham-irradiated mice. These results indicate that irradiation can inhibit the production of anti-dsDNA antibody related to SLE.

### 3.5. Irradiation Prolongs the Lifespan

**Figure 4:**
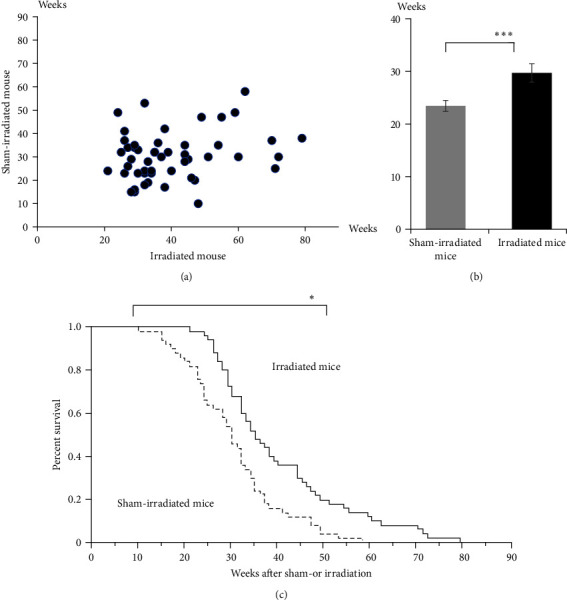
Irradiation prolongs the lifespan of NZBWF1 mice. (a) Comparison of the survival times in paired mice. The survival time indicates the duration from sham irradiation or irradiation to death (*n* = 50 paired mice, Group B). (b) Survival time of paired sham-irradiated and irradiated mice. Open and solid bars indicate sham-irradiated and 4 Gy-irradiated mice, respectively (*n* = 50 paired mice, Group B). (c) Survival ratios of sham-irradiated and irradiated mice (*n* = 50 paired mice, Group B). Blue and red lines indicate sham-irradiated and irradiated mice, respectively.  ^*∗*^0.01 < *p* < 0.05 and  ^*∗∗∗*^*p* < 0.001.

Next, the survival of mice was evaluated. The survival time of the irradiated mice (mean: 39.9 weeks) was markedly longer than that of the sham-irradiated mice (mean: 30.5 weeks) based on a comparison of the paired mice (*p*=0.0000803; Figure 4(a)) and the average of survival was also greater (*p*=0.0000673) (Figure 4(b)). The survival ratio was significantly higher in irradiated mice than in sham-irradiated mice (*p*=0.00144; Figure 4(c)). These results demonstrate that irradiation can prolong survival time.

### 3.6. Irradiation Reduces CD180-Negative Cell Percentages

**Figure 5:**
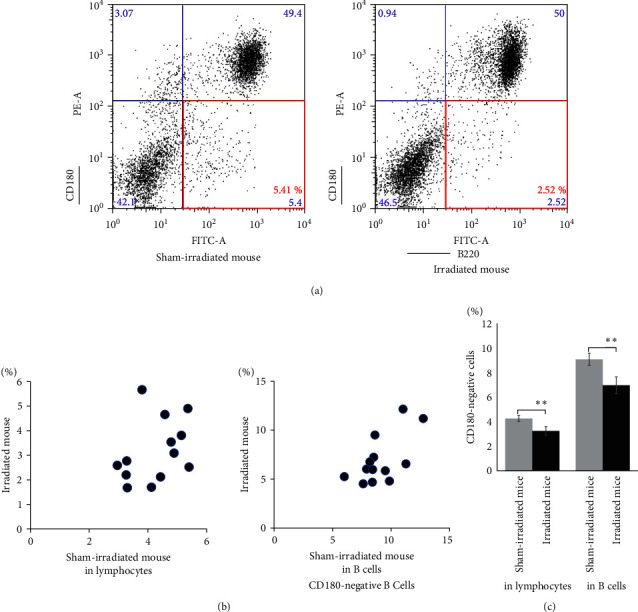
Irradiation reduces CD180-negative cell populations in NZBWF1 mice. (a) Flow cytometric analysis of CD180 expression in splenocytes from paired NZBWF1-irradiated (right figure) and sham-irradiated (left figure) mice 15 weeks after treatments (Group A). CD180-negative B cells are shown inside the red square. (b) Frequency of CD180-negative cells population within splenic lymphocytes (up) and splenic B cells (down; *n* = 13 paired mice, Group A). (c) CD180-negative cell population in the spleen. Open and solid bars indicate sham-irradiated mice and 4 Gy-irradiated mice, respectively (Group A). Each group consists of 13 paired mice. Data are presented as the mean ± standard error.  ^*∗∗*^0.01 < *p* < 0.05.

Previous studies demonstrated that CD180-negative cells are sensitive to radiation [22, 29], which is consistent with our data, as shown in Figure S1; those were performed using BALB/c mice *in vivo* and using splenocytes from NZBWF1 mice *in vitro*. The present study investigates the irradiation-induced effects on CD180-negative cells in NZBWF1 mice *in vivo*. The percentage of CD180-negative cells in the spleen was significantly lower in irradiated mice than in paired sham-irradiated mice 15 weeks after irradiation or sham irradiation using a flow cytometric analysis (Figure 5(a)). Moreover, irradiated mice had a markedly and significantly smaller population of CD180-negative cells than sham-irradiated mice among both the lymphocytes (3.17%, 4.25%, *p*=0.0088) and B cells (6.92%, 9.08%, *p*=0.0018) populations (Figures 5(b) and 5(c)). These results revealed that irradiation reduced CD180-negative cells.

## 4. Discussion

In the present study, we demonstrated that irradiation can effectively mitigate renal lesion grades, reduce urinary protein levels, and suppress autoantibody production in the peripheral blood, resulted in a significant extension of the lifespan of SLE model mice *in vivo*. Moreover, we showed that CD180-negative splenic cell populations were reduced by radiation treatment. These results suggest that irradiation can suppress the progression of SLE-like morbidity in NZBWF1 mice and that CD180-negative cells may contribute to the pathogenesis of SLE-like morbidities.

Renal injury is a critical factor of SLE that is directly correlated with disease burden and prognosis. In this study, histopathological assessment of renal damage in NZBWF1 mice revealed a markedly lower grade of renal injury in irradiated mice than in sham-irradiated mice 15 weeks after treatment. Moreover, this outcome was accompanied by suppressed proteinuria with time. These results are consistent with the findings of time course studies on untreated NZBWF1 mice, which showed increased urinary protein levels with aging [39]. In fact, the duration from disease onset to level 3+ proteinuria (approximately 300 mg/dL protein) was markedly longer in irradiated mice than in sham-irradiated mice, indicating a longer lifespan and better quality of life after irradiation. Hence, irradiation may be an alternative strategy to prevent renal damage and improve the overall outcome of patients with SLE.

A previous study demonstrated that the levels of anti-dsDNA antibodies increase with the development of SLE-like morbidity in NZBWF1 mice [39]. Similarly, we found that the anti-dsDNA antibody levels were lower in irradiated mice than in sham-irradiated mice at all time points tested (6, 12, 15, and 36 weeks after treatment). Anti-dsDNA antibody is highly specific for SLE [42, 43], reflecting the SLE disease activity index [24, 25]; thus, the overall goal of SLE treatment is to reduce autoantibody levels [44]. Therefore, the present findings demonstrating the *in vivo* regulatory role of irradiation on anti-dsDNA antibody levels provide a new insight for therapeutic strategies for SLE.

Indeed, this inhibition of autoantibody production may have contributed to the reduced SLE-related renal lesions observed in NZBWF1 mice, since the autoantibody accumulation can cause inflammatory lesions in various organs, including the kidneys. This result is consistent with previous reports showing that decreases in anti-dsDNA antibody levels are associated with amelioration of glomerulonephritis in NZBWF1 mice [45, 46].

Moreover, the present data provide additional evidence that CD180-negative cells can be modulated by radiation therapy. The reduced population of CD180-negative cells may have resulted in the inhibition of anti-dsDNA antibody production, as these cells have been reported to produce autoimmune antibodies, as these cells have been reported to produce autoimmune antibodies, such as anti-dsDNA and anti-histone antibodies [39].

Total lymphoid irradiation has been reported to prolong the survival, reduce proteinuria, and decrease anti-DNA antibody levels in NZBWF1 [17, 47, 48] and MRL/1 mice [18], but the mechanisms underlying these effects remain to be clarified. These reports implicated that the immune system was concerned with the alleviation of SLE-like morbidities. Although there is a possibility that other immune system cells or molecules may be involved in these processes as a result of irradiation, all collected data suggest a strong relationship between the regulation of CD180-negative cells by radiation and SLE symptom amelioration.

Novel biologic agents targeting B and T cells and relevant cytokines have been proposed for the treatment of patients with SLE [48]. Indeed, therapeutic strategies that target B cells via surface antigens and survival factors can effectively eliminate B cells; however, nonspecific deletion of B cells has a negative impact on normal immune systems [17, 49]. Therefore, strategies that specifically target pathogenic B cells are important [19, 48, 50]. CD180-negative cells are regarded as a therapeutic target in patients with SLE because they have been implicated in disease development through the production of autoantibodies [51, 52]. Nonetheless, clinical trials targeting CD180-negative cells have been challenging due to the lack of an effective marker for these cells. Studies to identify additional molecules associated with CD180-negative cells are underway [52, 53]. The difficulty of targeting CD180-negative cells via means other than radiosensitivity is again a limitation of our study.

Although whole-body radiation exposure can have harmful side effects, targeted spleen irradiation may represent a better option with fewer side effects. In this study, irradiation at disease onset demonstrated long-term therapeutic benefits; however, its underlying mechanisms remain unclear. One possibility for this mechanism is that pre-stage CD180-negative cells may be more radiosensitive than mature CD180-negative cells. Additional studies should explore whether naïve CD180-negative cells are more radiosensitive than mature CD180-negative cells and for how long the effects of radiotherapy prevail in the body. Further experiments are also needed to investigate the involvement of other immune system cells in SLE and their sensitivity to radiotherapy.

## 5. Conclusion

In SLE, autoimmune antibodies are important factors contributing to symptom onset and exacerbating the disease course. Suppression of autoantibody production is, therefore, an important treatment strategy for SLE. The present study demonstrates that irradiation can suppress autoantibody production, prevent renal damage, decrease proteinuria, and ultimately extend the lifespan of NZBWF1 mice showing SLE-like morbidity. CD180-negative cells are the most likely cells involved in this effect. Based on an SLE preclinical model, the results presented herein provide new foundations for the development of therapeutic strategies to eliminate autoantibodies and address SLE development *in vivo*.

## Data Availability

Data are available from the corresponding author upon reasonable request.

## Supplementary Materials

Supplementary Materials Figure S1: CD180-negative cells are radiosensitive compared with CD180-positive cells.  ^*∗∗*^0.001 < *p* < 0.01,  ^*∗∗∗*^*p* < 0.001.
