# Supplementary material for: Irradiation Attenuates Systemic Lupus Erythematosus-Like Morbidity in NZBWF1 Mice: Focusing on CD180-Negative Cells
Source: J Immunol Res. 2023 Oct 18;2023:9969079. doi: 10.1155/2023/9969079 (PMC10599955; doi:10.1155/2023/9969079)
Supplement: Supplementary Materials — Figure S1: CD180-negative cells are radiosensitive compared with CD180-positive cells. ∗∗0.001 < p < 0.01, ∗∗∗p < 0.001. [file 9969079.f1.pptx]

## Slide 1
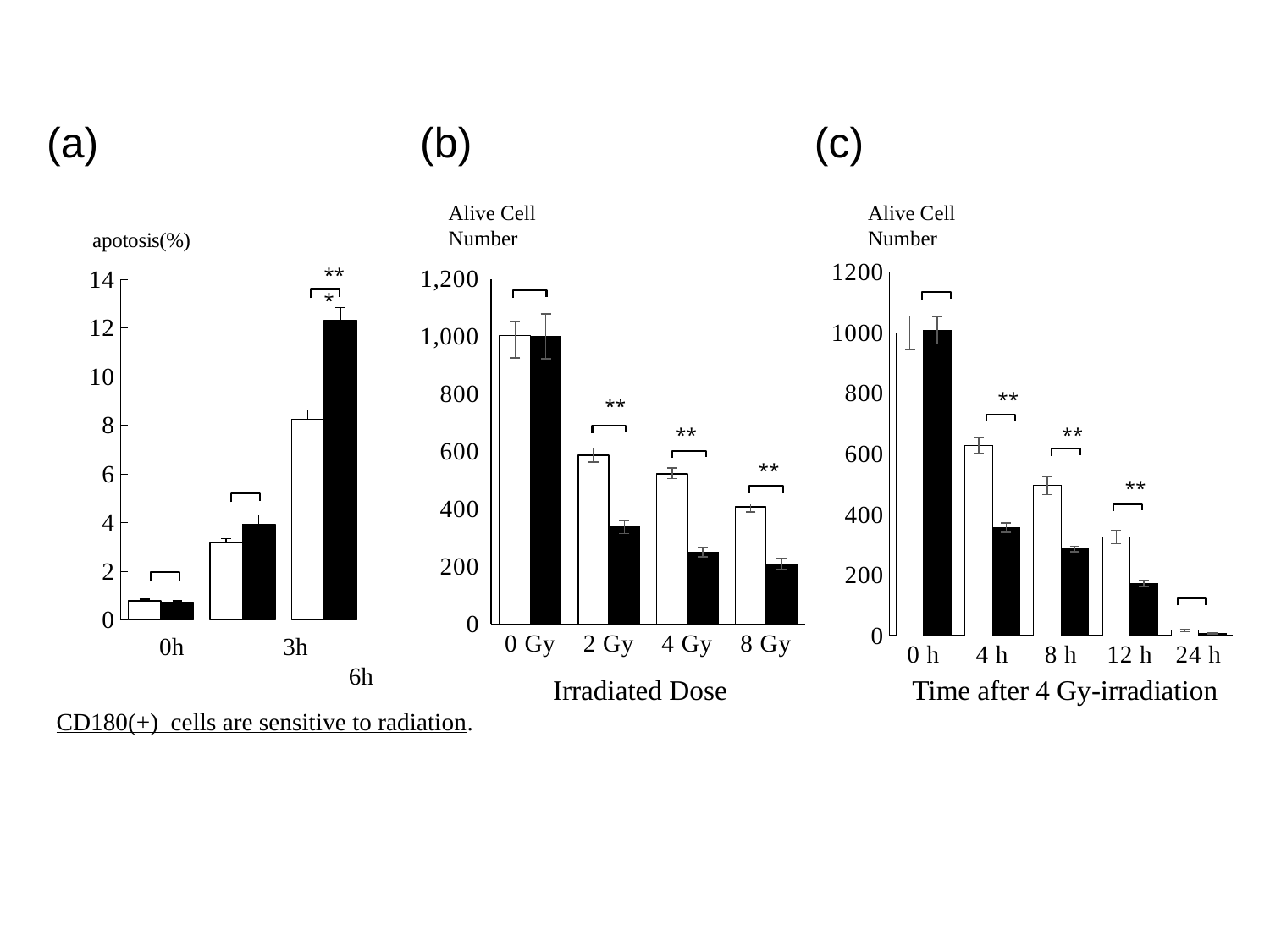

(a)
(b)
(c)
Alive Cell
Number
Alive Cell
Number
### Chart
| Category | | |
|---|---|---|
| 0 h | 0.78708321127251 | 0.718691807628847 |
| 3 h | 3.1687321523229 | 3.930191577577173 |
| 6 h | 8.247815921316695 | 12.312915755176 |
### Chart
| Category | CD180(+) alive cells | CD180(-) alive cells |
|---|---|---|
| 0 Gy | 1002.85 | 1000.0 |
| 2 Gy | 587.1137409598948 | 337.60683760683764 |
| 4 Gy | 522.079772079772 | 249.99999999999997 |
| 8 Gy | 406.69515669515675 | 209.40170940170938 |
### Chart
| Category | CD180(+) alive cells | CD180(-) alive cells |
|---|---|---|
| 0 h | 1000.0 | 1008.7719298245613 |
| 4 h | 627.7412280701755 | 357.14285714285705 |
| 8 h | 495.2485380116959 | 286.4583333333333 |
| 12 h | 325.23616734143053 | 172.6973684210526 |
| 24 h | 17.712550607287447 | 7.881030701754385 |0h　　　 3h	　 6h
Irradiated Dose
Time after 4 Gy-irradiation
CD180(+) cells are sensitive to radiation.
